# Understanding the User-Generated Geographic Information by Utilizing Big Data Analytics for Health Care

**DOI:** 10.1155/2022/2532580

**Published:** 2022-10-06

**Authors:** Hidayat Ullah, Alaa Ali Hameed, Sanam Shahla Rizvi, Akhtar Jamil, Se Jin Kwon

**Affiliations:** ^1^Faculty of Engineering and Natural Sciences, Department of Computer Engineering, Istanbul Sabahattin Zaim University, Istanbul, Turkey; ^2^Department of Computer Engineering, Istinye University, Istanbul, Turkey; ^3^Raptor Interactive (Pty) Ltd, Eco Boulevard, Witch Hazel Ave, Centurion 0157, South Africa; ^4^Department of Computer Science, FAST School of Computing, National University of Computer and Engineering Sciences, Islamabad, Pakistan; ^5^Department of AI Software, Kangwon National University, Samcheok 25913, Republic of Korea

## Abstract

There are two main ways to achieve an active lifestyle, the first is to make an effort to exercise and second is to have the activity as part of your daily routine. The study's major purpose is to examine the influence of various kinds of physical engagements on density dispersion of participants in Shanghai, China, and even prototype check-in data from a Location-Based Social Network (LBSN) utilizing a mix of spatial, temporal, and visualization methodologies. This paper evaluates Weibo used for big data evaluation and its dependability in some types rather than physically collected proofs by investigating the relationship between time, class, place, frequency, and place of check-in built on geographic features and related consequences. Kernel density estimation has been used for geographical assessment. Physical activities and frequency allocation are formed as a result of hour-to-day consumption habits. Our observations are based on customer check-in activities in physical venues such as gyms, parks, and playing fields, the prevalence of check-ins, peak times for visiting fun parks, and gender disparities, and we applied relative difference formulation to reveal the gender difference in a much better way. The purpose of this research is to investigate the influence of physical activity and health-related standard of living on well-being in a selection of Shanghai inhabitants.

## 1. Introduction

Location-Based Social Media Networks (LBSNs) have come a long way since we first took a leap in 2007 when Facebook opened up for use. The LBSN model is one that has been widely used for a variety of purposes including commercial, governmental, and nonprofit works. Though location-based social networking has still been regarded as an innovation, the paradigm is rapidly expanding. People have been taking advantage of the location-tracking data and the resulting insights to build more effective social applications for marketing, advertising, commerce, and even to help in the discovery of real-world phenomena. To date, over one billion people around the globe have joined social networks, and many of these social networks do not use geocoding to get their information. Instead, they often rely on manually curated lists of user-submitted location identifiers. As a result, a wealth of new information is being generated each day from such a model, and researchers are beginning to mine these records in a systematic way.

The input is typically supplemented with facts, visuals, geo-locations, and textual data, which would be used to make more research on many aspects of people's actions. Past studies used either actually acquired fact for groups in specific classes, including such leisure or LBSN data for the entire society with no preset deployments. If properly classified, the numerous aspects of the LBSN facts would prove to be a powerful resource of information for assessing people's activity in a variety of fields like as entertainment, education, tourism, dining, and aviation.

As a result, in this research, we will fill the gap in research of employing LBSN statistics in article or content by assessing which amusement places Shanghai citizens desire to attend. Several studies have been conducted in an attempt to analyze and imitate human actions through geo-data. The latest research, for instance, leverages check-in records from globally prominent LBSNs such as Twitter, Facebook, and Foursquare to reveal connections and trends among consumers such as gender, expert or less skilled groups, and age groupings [1–3].

Based on the check-in time, the user may have spent some time at a certain place. This is a potential feature of spatial and temporal aspect. Some spatio-temporal features are related to the check-in time, longitude, latitude, location difference, and check-in time difference. In these features, we can only retrieve information for a location, and the time difference is very small. If we set the threshold of the check-in time difference, then we can select check-in data of different timestamps in the same location. The check-in time can help us to know where a person spent her/his time, and the check-in time difference can help us to understand the daily movement patterns of each user. However, this information does not help us to understand the time spent at different locations or the movement paths in LBSN.

Other researchers, to our knowledge, did not incorporate this earlier. As per significance, we focused on three unique parts of investigation on Weibo's check-in data from the city of Shanghai for two years, from July 2015 to June 2017, to identify spatio-temporal patterns and inhabitant's predictions utilizing physical engagement sites and density prediction. As a consequence, three key aspects of the assessment are highlighted in the newest study. Our input to the existing study is on these topics:

- Time variations of an hour, a week, and a daytime
- Data collection and physical action site study
- Using spatial analysis to model and predict density

Section 2 of this study contains relevant material on big data, LBSNs, and the important significance in a range of sectors and also articles on Weibo, Shanghai, and China.  Section 3 summarizes both the dataset and the analytical approach.  Section 4 contains the facts and a narrative, while Section 5 contains the study's findings and suggestions.

## 2. Related Work

A big issue that we should consider is the question, where do we find the “Big Data?” Actually, even big data analysis does not exist without facts although this does not mean that big data has to include all the data, and sometimes, some data may be more important. In some areas, particularly social sciences, “Big Data” involves massive open online courses (MOOCs), blogs, wikis, video recordings, live tweets, live chats, microblogs, video images, and documents that capture data (i.e., digital documents, websites, images, and videos) in a manner that enable the monitoring and management of these data over time and space, and this term is more often associated with Internet data and specifically Internet Big Data although the term “Internet Big Data” can also be applied to traditional data sources too. However, Big Data is not to be considered the opposite of “small data,” with the latter referring to the data that is more easily handled and analyzed [4–8]. Instead, it is the integration of and the interrelation between small and large datasets.

Big Data is a type of data, collected and processed in a structured and unstructured way. On the one hand, a Big Data application is focused on collecting data that is big, complex, and diverse. On the other hand, another Big Data application has a focus on transforming the collected data into information and knowledge. Ovadia demonstrated and emphasized the importance of “Big Data” for intellectuals and social scientists, claiming that “Big Data” is too important to overlook since many social scientific study needs a large amount of facts and enormous datasets [9].

Several research fields, including time and human mobility, space geography, human behavior, and metropolitan activities, originated with statistical data collected via surveys, visit records, questioners, interviews, and other hand-crafted datasets [10, 11]. These methodologies comprising geo-information, have recently spread powerful resources of information for such scientific papers, [6, 12, 13]. Tracking users' movements and actions have become simple because of the fast progress of mobile technology and the extensive use of mobile devices [14]. Though giving an approximated space just next to the mobile's base transmitter in which the calls were routed, the dataset demonstrated competence in forecasting user positions with little time, which was then used to anticipate user activities [15].

Zhu presented numerous GIS (geographic information system) components and their importance in pattern extraction and municipal studies by demonstrating how to evaluate and display the spatio-temporal parts of reusable garbage, collection, and rehabilitation [16–18]. The authors in these researches utilized these data for health purposes [19–25] and medical data security [18, 26, 27]. These researches are based upon endoscope image, medical image registration, and soft tissue modeling [28–30].

The term “business intelligence” (BI) first appeared in the late 1990s [31], At the organizational level, “Big Data” is a significant area. Prediction, ad hoc inquiries, and aggregation-based reporting, along with processing organized and unorganized data and combining “Big Data”-based systems, all contribute to better decision-making [32–34]. The BI systems include data warehouses for gathering clean, precise, and comprehensive data from a variety of origins, and also online analytical processing (OLAP) for real-time multidimensional analysis with processes, for example, combining, screening, roll-up, spin, and exploration for details [35].

OLAP is such a well-recognized and well-respected methodology for “Big Data” research in BI systems [36]. However, while BI, OLAP, and data warehouse are powerful tools for coping with vast volumes of data and a vast variety of operations, they also provide a challenge owing to the considerable cost, space, and computing resources necessary [37–40]. The authors introduced new data mining techniques in these researches [41–43]. Digital social networks have been demonstrated to be the most significant source of “Big Data” to research personal activity since they have been used and are fast expanding in virtually every location across the world.

Customers of the LBSNs' digital services may upload and exchange their actions, preferences, and whereabouts, resulting in massive amounts of data for numerous researches on many areas. Papers [44, 45] go into excessive details regarding the human behavior analytic techniques. Lindqvist [46] discovered the usage of LBSNs, which was encouraged by earlier research studies; for example, practical studies and socio-spatial aspects utilizing LBSNs [47] and a personalized geo-social suggestion relied on a dataset from two independent LBSNs, namely,Gowalla and Foursquare [48,^49^]. By gathering regular users at various locations, researchers in two UK cities used comparable check-in data from LBSN to enhance the recommendation algorithms.

Li [50] did a thorough examination of location-based data from 2.4 million places in 14 states to determine the elements affecting place popularity. The research's findings identified three major factors that influence a location's fame: site profile, site age, and site type. Another study on consumer actions at numerous venue categories focused on “Food” in Riyadh, Saudi Arabia, and revealed that when clients attend food venues, they seem to be more receptive to discussing their behaviors. The check-ins of about 19,000 Swarm (Foursquare) members from three metropolitan centers, mainly San Francisco, Hong Kong, and New York were utilized to evaluate linkages between different sites at different times of the day [51].

Several researches have been conducted all across the universe to investigate different consumer and check-in characteristics utilizing LBSN sources of information like Twitter and Foursquare. These traits have been used in a variety of sectors, together with transportation trends, venue categorization, and urban development and growth [52]. Weibo, a well-known Chinese LBSN, was used in this investigation and demonstrated to be beneficial. In research for Shenzhen [53], Weibo data were utilized to analyze the request powers of tourist charms. Another study on people's action trends and engagements was assumed in order to observe urban boundaries in Beijing [54]which likewise used Weibo check-in. In a comparable vein, Shi et al. [52] utilized Weibo data to examine attributes of tourism sites based on numerous details provided by the LBSN, and the evaluation was combined with feelings from user reviews. Wu et al. [55] performed spatio-temporal assessment grounded on the period of day and the variance in check-in patterns between weekdays and weekends [56, 57] also examined check-in research containing 21 of Wuhan's most famous lakes.

## 3. Dataset and Methodology

### 3.1. Study Area and Data Source

**Figure 1:**
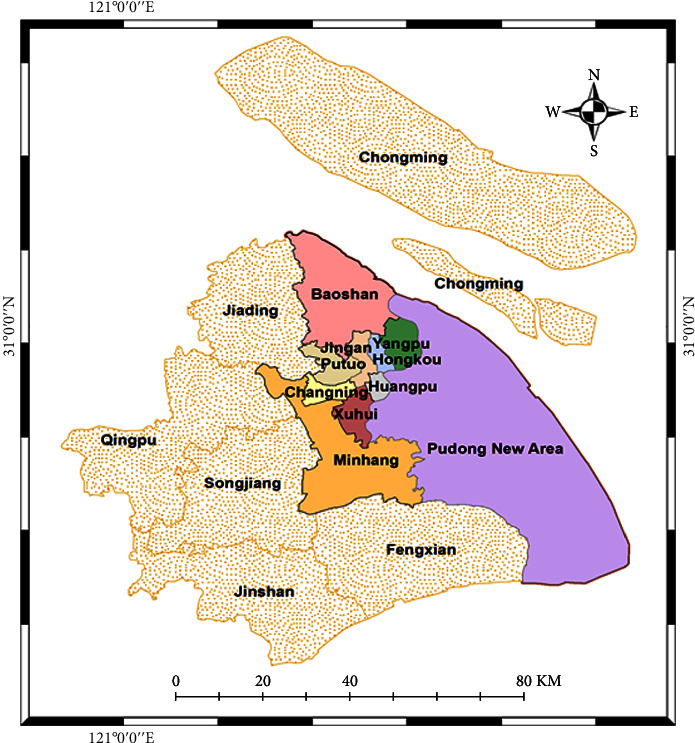
Study area.

Shanghai, like the other megacities in East Asia, experiences urban sprawl. This is particularly the case in low- and middle-income groups and among minority groups, such as the ethnic minorities (like Zhuang, Dong, Miao, and Hui), while the rich and high-income groups have higher purchasing power are more likely to move into the inner city and tend to be concentrated in the central city. The hour of the day and the difference in check-in trends across weekdays and weekends were used to do a spatio-temporal evaluation.  Figure 1 displays the research domain, and it can be seen that we focused and utilized the data of 10 districts.

The data are gathered from Weibo, which contains people' check-in details while they are at a specific place. The check-ins from Weibo contain latitude, longitude, accuracy (e.g., 1.8 meters), address, time, and content. In addition, Weibo has been updated more than 500 million times in a day, covering about 1/5 of all of Weibo's activity, which makes it one of the most extensive and authoritative datasets in the field of big data. The spatio-temporal check-ins gathered in this research is collected from the city of Shanghai, which covers four prefectures. There are three major problems associated with collecting the spatio-temporal check-ins of the Weibo from a single city. First, it is very difficult to extract real locations from a large number of posts with various locations. Thus, we have to use heuristics such as the latitude and longitude of the home location to extract places. Second, as Weibo user accounts contain a location-based tag, the real location of the user is more or less accurate, but the real location of posts is different from the location of the corresponding Weibo users. Third, some posts just contain a location without other information such as a picture. The number of visits has risen to 500 million by the end of 2018, with 462 million monthly regular individuals and 200 million daily active users. This study focuses on two years of socially collected spatio-temporal check-ins from Weibo in Shanghai, from July 2015 to June 2017.

The fundamental reason for utilizing LBSN is to exchange activities and observations, which results in the establishment of a new close social fellowship group. This allows experts to deduce a broad spectrum of individual action and pleasure from the geo-data collected by these LBSNs. This study's data originated from LBSN's Weibo account. We utilized the Weibo API (application programming interface) based on Python to collect data from check-ins in Shanghai.

**Figure 2:**
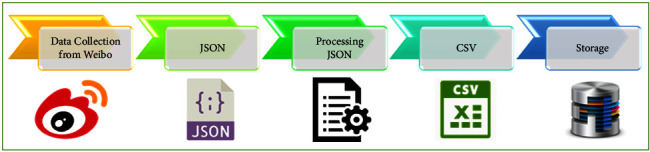
Process flow of data.

This was compiled in 2017 within over 3.5 million cumulative check-ins from approximately two million people. For the current study, the data were converted from JavaScript Object Notation (JSON), the standard API Java programming language, to comma-separated values (CSV) using Mongo DB. The information processing route is depicted in Figure 2.

JSON is a tiny data transfer standard that uses human-readable language to transmit data entities, while Java is an object-oriented programming environment [58]. The information was combined into one file in the CSV (comma-separated values) format for more processing and analysis with the tools indicated.

**Figure 3:**
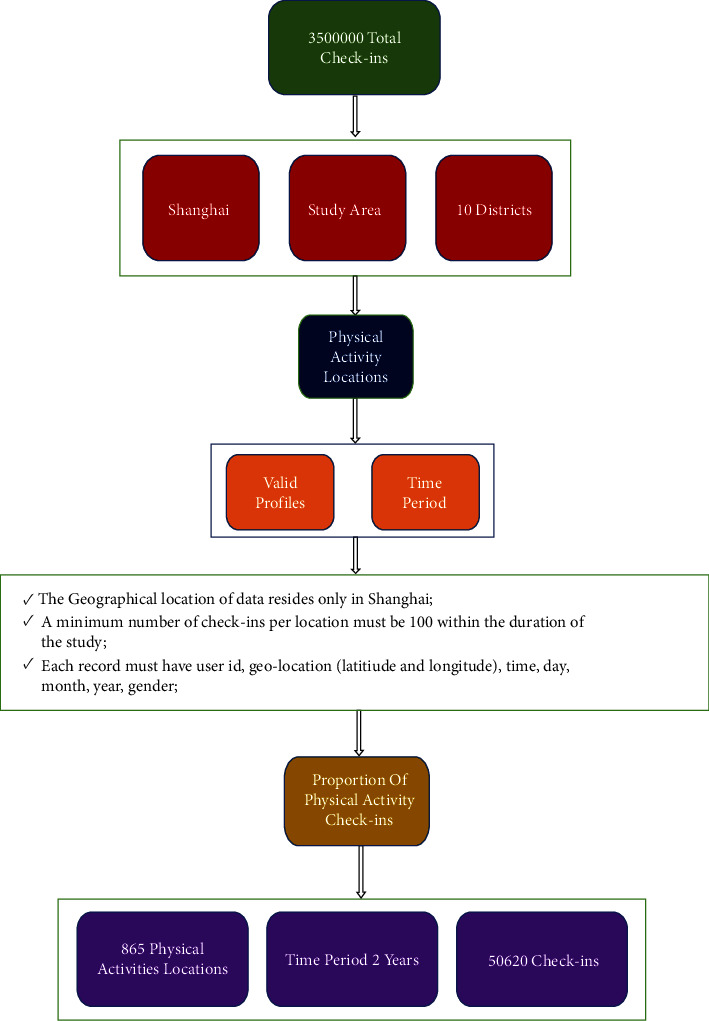
Criteria of research.

All of the contributors' information, including geolocation, might be recorded in a database. We collected the data in the CSV format and then used a criterion to determine the significance of the results. The criterion figure is depicted in Figure 3.

The CSV standard is the most prevalent and significant standard for databases and spreadsheets, and it uses commas as delimiters for different parameters. In the JSON file standard, keys (ID, latitude, longitude, etc.) are used as headers for the CSV file format, while values (5404478798, 121.544449, 31.268159, etc.) are used as descriptive data. In the CSV file, Table 1 shows an illustration of a “check-in.”

### 3.2. Social Media Data Analytics Framework

**Figure 4:**
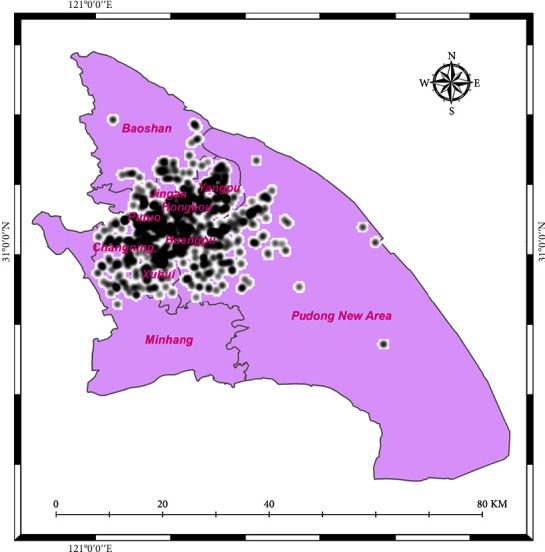
Check-in density of activities.

**Figure 5:**
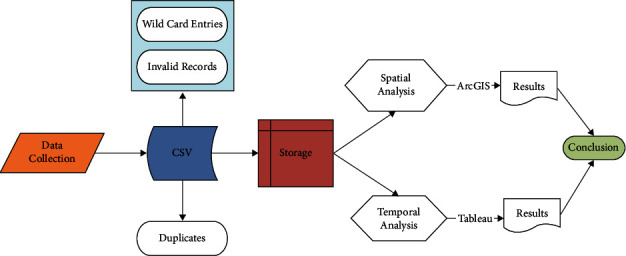
Research methodology.

A social media data analytics framework was constructed to provide the analytical capability of a business unit or an enterprise organization that needs to use the social media to analyze the social media data in their systems. Social media data are the social media messages, tweets, blog posts, pictures, and so on created by the user(s). Social media data analytics framework can analyze this data, process them, and provide the required information. Social media data analytics tools are tools that perform real-time data analytics and report on social media. They are used to analyze the social media data, discover valuable insights about social data, and deliver actionable recommendations. Our broad geographical assessment approach is depicted in Figure 4. The first element is broken into two parts: data collection (downloading Weibo data) and data cleaning. Following that, the LBSN data is analyzed , as shown in Figure 5.

We used ArcGIS software for the spatial analysis and tableau for the graphical representation of temporal analysis.

### 3.3. Spatial Method

KDE is used to estimate the distribution of data and can be useful in estimating probability distributions of numerical variables and to calculate a density-based visual perception in a given image [47, 48, 52, 56, 59–68]. The method works by determining an exact probability distribution function in a given image and then calculating the integral of this probability distribution function to obtain corresponding intensity estimation. There are a number of different estimation techniques for numerical variables. The most frequently used methods are the kernel density estimate (KDE), the Parzen window (PW), and the Epanechnikov window. As the name indicates, Kernel density estimate uses the kernel function as its basis for a density estimate. The Parzen window method is a variation of KDE that uses a Gaussian kernel function. The Epanechnikov window technique is a nonparametric density estimation approach that makes use of the Epanechnikov window's flattening capabilities. Since the 1960s, the Epanechnikov window has been utilized as a nonparametric concentration prediction approach. However, the KDE and PW methods are parametric methods. The Epanechnikov window method is a nonparametric method. Parametric methods are based on distribution functions. To compute a parameter estimate using a parametric method, it is necessary to specify the parameter values. Parametric methods are also commonly used in image analysis and computer vision. A parametric method assumes that the object that is to be described has a unique parameter. Parametric density estimation is widely used to estimate the parameters in the distribution of physical quantities and to estimate probability distributions.

We utilized the KDE method here in our research because for modeling spatial-densities, the KDE method has also been used in fields such as health, marketing, and environment [48, 60, 61]. Recently, as it is used in the analysis of spatial data for determining distribution of the phenomena in geospatial data, many researchers applied the technique to this field. One of the basic topics is that the density of points is determined, but the points are distributed on grids due to the data structure. There are many methods of determining the density. If the grid size is large, many points have been concentrated in each grid, and the points are not sparsely distributed. Thus, the density of the points is high. Thus, it is thought that the density is determined by using the density of the grid. This process is called density-grid transformation.

KDE has been used in studying the patterns of visitors in green parks [66, 69–77]. These studies, mostly concerned with the visitors' number, are based on the assumption that people who visit a green park have similar behavior. To identify the number of people who visit a green park, the visitors are often modeled using kernel density estimation. The number of visitors in this method is simply defined by the area of each kernel. The mean of these individuals is called the mean estimator of the total number of people who visit a green park. It is not known whether this method gives an unbiased estimate of the number of people who visit a green park.

Let *E* be a set of historical data where *e*^*j*^=<*x*, *y*> is the geo-coordinates of a location, 1 ≤ *j* ≤ *n* for an individual *i*, *h*_*j*_ is the Euclidean distance to *k*-th near neighbor *e*^*j*^ in the training data. The KDE is stated as follows:

(1) $$\begin{array}{c} f_{KD}\left(e|E\right)=\frac{1}{n}\overset{n}{\underset{j=1}{\sum }}K_{h^{j}}\left(e,e^{j}\right)\text{,}\\ K_{h}=\frac{1}{2\pi h}\exp\;\left({-\frac{1}{2}\left(e,e^{i}\right)}^{t}\overset{-1}{\underset{h}{\sum }}\left(e,e^{i}\right)\right)\text{.} \end{array}$$

## 4. Results

**Figure 6:**
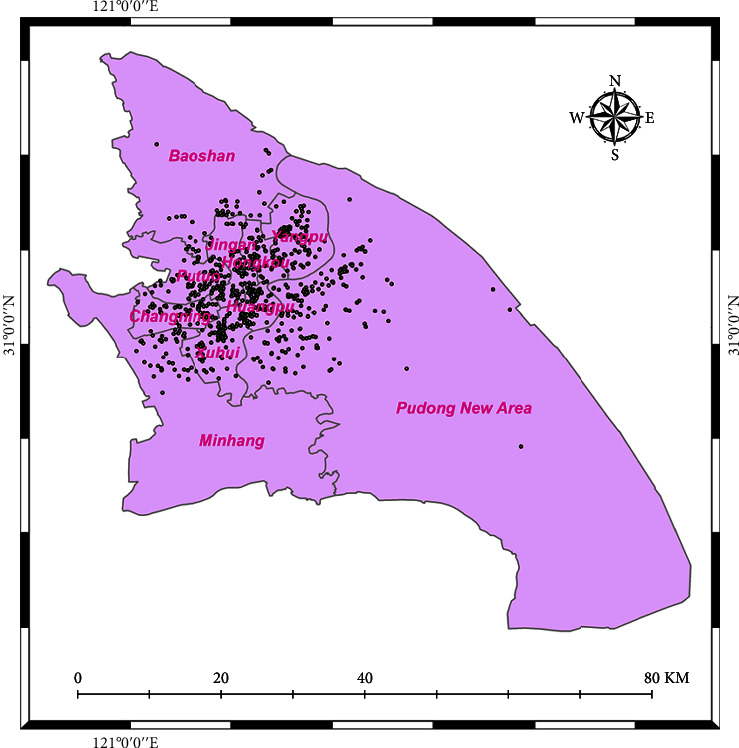
Locations of activities.

Shanghai City, with such a population of 22,125,000 residents and a land area of 4,015 square kilometers, has become one of the world's fastest-growing cities [78, 79]. Over two years, data have been accumulated on amusement check-ins. Every single check-in was allotted a value that best suited the physical activities taken out at that place, like gym, sports, and park exercise.  Figure 6 depict the total locations of such activities in our study area, and the total number of locations is 865, as can be perceived in the given figure.

We investigated the geographical variation of check-in data with KDE and showed the Weibo geolocation check-in dataset with ArcGIS. From July 2015 to June 2017, the overall check-in intensity in Shanghai was depicted in Figure 4. Sections highlighted in black represent a bigger number of persons, a higher frequency of activity, and a better knowledge of social network usage. It is just not surprising that the seven districts look thicker than the other three districts even though the three districts have a bigger area.

**Figure 7:**
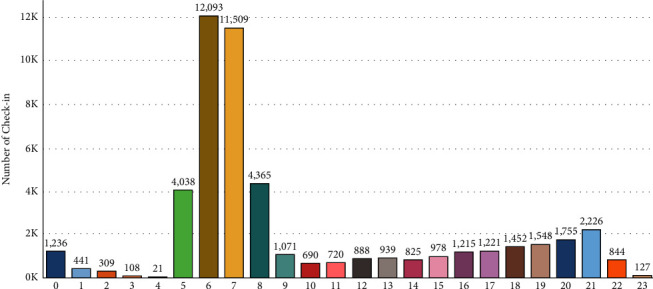
Hourly check-in frequency.

Figure 7 demonstrates the temporal fluctuations in the number of visitors over 24 hours. Even though visitors participated at all hours of the day, most check-ins were recorded between 05 PM and 09 PM among the entertainment sites investigated. Until midnight, the tendency will continue to rise.

**Figure 8:**
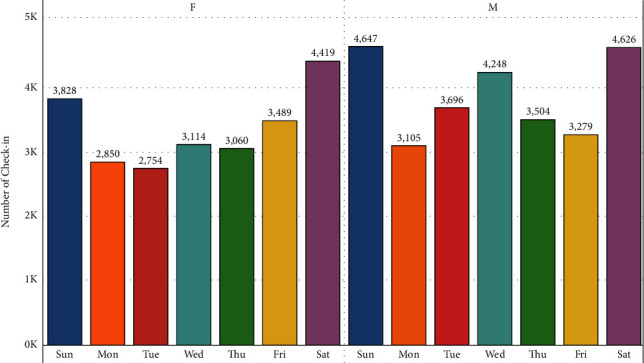
Daily number of check-in and gender difference.

Figure 8 illustrates the digit of check-ins for each day with the gender difference, and it can be observed that weekends have a bigger size of check-ins than weekdays but the number of males is higher than that of females for each day, and it is shocking.

**Figure 9:**
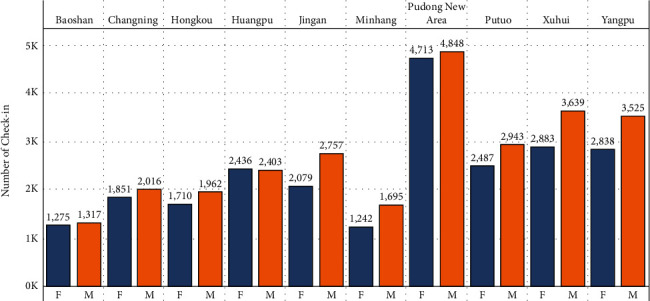
Check-ins distribution in districts.

Check-ins are disparaged at the district level to provide a more accurate picture of entertainment place distribution in Shanghai City.  Figure 9 shows that the distribution of check-ins is the greatest in the Pudong region, preceded by the Huangpu district.

This pattern can be explained by the fact that the Pudong new area district is larger than some other districts. Another factor to bear in mind is that check-in dispersion is stronger in the city region than in the outskirts. Gender differences may also be evident, with male check-ins outnumbering female check-ins throughout all areas.

**Figure 10:**
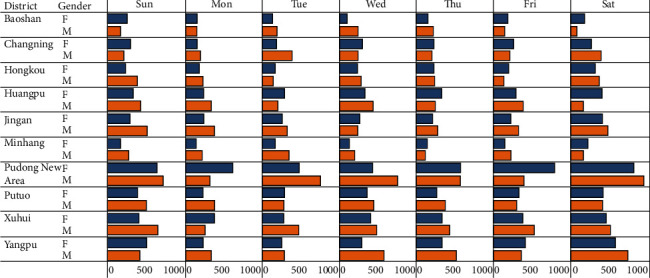
Overall statistics.

Figure 10 illustrates the total overall check-ins made each day in all the districts of study area. It can be witnessed that if it is the day or a district, the number of check-ins made by men is higher than that of females.

We applied an analysis on our given data for revealing the comparison of gender difference in the physical strain activities which are really necessary for a healthy life.  Table 2 shows the percentage of number of check-ins.

To compare districts and days, we used a relative difference (*d*_*r*_) of a given gender. When there is a big difference, the absolute difference is small. If the difference between females and males is large, the relative difference is smaller. For example, we calculate the difference between total male check-ins and male check-ins per day in weekdays in the districts. There are some districts with a relatively large difference, and there are some districts where the difference is small. It is generally used as a quantitative pointer of quality control and quality assurance and is stated as follows:

(2) $$\begin{array}{c} d_{r}=\frac{\left|P_{m}-P_{f}\right|}{\left(\left|P_{m}\right|+\left|P_{f}\right|/2\right)}\text{.} \end{array}$$

Finally, the analysis examines the difference between all-male group and female-male group in Shanghai and the time span of two years to form male-only groups and female-only groups. In this context, all-male group means that the total check-ins of male is more than that of females, whereas the female-only group means that females' check-in is more than males' check-in. Tables 3 and 4 shows that there are significant differences among gender in both days in week and districts. The results show that the frequency of women in the districts is less than the men in all days of the week, except Friday. This indicates that male users prefer other days to Fridays while women users are more active on Friday.  Table 3 shows the difference between male and female user check-ins on the different days of the week. As expected, we see significant difference among the days of the week. Overall, Saturday has the highest number of male check-ins, and Friday has the highest number of female check-ins. This indicates that different days of the week are equally important in terms of check-ins. However, we see some differences in the number of check-ins among the different districts.

We see some differences in the number of check-ins in the different districts.  Table 4 shows that districts Jingan, Putuo, Xuhui, and Yangpu have a significant difference between male and female check-ins. These results confirm the previous observation that there is difference in the distribution of the male and female users in the city.

## 5. Discussion

The study of urban environment is attracting increasing research interest and has developed rapidly in recent years. However, there is still a gap between academic and popular knowledge about activities, and most existing research are conducted in the form of case studies in one or two places. Most of the research data and conclusions are based on the investigation of one city or district. The research results have limited significance for the whole city. However, most study data are examined using statistical approaches that do not take into account spatio-temporal features, so they cannot represent the popularity of activity across time and location. This not only contradicts the features of the urban area but also fails to suit the demands of modern city design. In this paper, we aim to analyze the characteristics of the popular physical activities in Shanghai from a spatio-temporal perspective and compared the gender difference. We study how the Shanghai public's popularity for different kinds of physical activities has changed over time, which popular location is most favored by the public, and in which places the physical activity is most favored. The data of Shanghai Weibo, the largest social media platform in China, is used to conduct the investigation. In summary, we find those as follows: (1) There are some places with higher popularity compared to others and (2) there is also difference in the popularity of physical activities over time and space. We believe that these findings can not only provide references for urban management but also help to understand the public's favorite locations in one city.

Several limitations exist in this study. Data security is really necessary for dataset or any method [80, 81]. First, since the data we collected were based on online records, the results may be biased because different Internet access habits among different geographical areas affect the data. For example, people in rural areas may have more Internet access and thus have more chances of accessing social media than people in urban areas. Second, we only selected a single city as the sample, and this limited the generalization of our results. Third, although WeChat check-in data is a very powerful tool in the evaluation of physical activities quality, this approach still contains subjective information, which cannot be quantified. Therefore, the evaluation of data quality in our study should be revised based on more and more data-driven evaluation methods.

Accessibility of the data is a major hurdle to LBSN research due to data and confidentiality safety considerations. The ability of LBSNs to reveal users' and their contacts' present geolocation which raises serious privacy concerns. They are worried about their privacy, so are administrative or business users that communicate data over LBSNs. Personal information is sometimes given freely or inadvertently. Though the data are acquired frequently by offering customers special rights and prizes in exchange for their information, this is not at all times the case.

Our findings indicate that this new channel has been relatively successful in terms of both social media check-in and activity tracking activity. However, despite this success, this channel only covers roughly half of all locations. For this reason, our findings have important implications for how location-based data might be used. In terms of data validity, the study provides a unique opportunity to compare Weibo check-ins with activity tracking. Only a few studies have compared the two channels. As with other research, we found that the Weibo data were accurate, but not complete. As discussed in the introduction section, we believe this is not a reflection of the data validity but rather an aspect of the usage of these services.

Our findings contribute to the understanding of user behavior and the understanding of the behavior of social media users in general. Because check-ins are executed by a moderately small group of Weibo users, they have been used in other studies as a good pointer of Weibo users' day-to-day activity [71] However, our findings reveal that the number of locations has not been increased significantly in Shanghai. When compared to prior studies, it can be noted that all of the studies show that female consumers are much more active than males during the various activities in Shanghai [69–74, 82, 83].

## 6. Conclusion

We looked at consumers' check-ins in 10 distinct districts of Shanghai, focusing on different aspects of geo-referenced data. We utilized the KDE method by collecting the check-ins of different users of Weibo microblog and all the check-ins were collected during the users posted when they were doing any physical activity in gyms, playing grounds, and other parks as well. Our data shows that most people submitted their physical action check-ins in Shanghai's city center, which is separated into seven districts. Pudong new area and Huangpu are denser districts than others, and weekends have more check-ins compared with other days. The conclusions reveal that male users are more active in physical activities either in gym or playing ground in all the districts of Shanghai and its shocking revelation. The purpose of our research is to create awareness among people about the benefits of physical activities on health. The research may be valuable in detecting more overcrowded areas in Shanghai so that regulating or management institutions can more efficiently watch and aid such districts, notably in events, public action, and urban development, among other things.

## Figures and Tables

**Table 1:** Example of Weibo check-in.

Building_id	User_id	Date	day	Time	Year	Gender	Lon	Lat	Address
2930091850	### ^*∗*^	24	Wed	0 : 00 : 03	2016	F	121.58689	31.2108	Sports and leisures
3785887251	### ^*∗*^	11	Fri	1 : 24 : 45	2016	F	121.3710578	31.14217173	Sports and leisures
3949989322	### ^*∗*^	29	Mon	3 : 02 : 19	2017	M	121.44087	31.18104	Sports and leisures

**Table 2:** Overall statistics.

Gender	District	Sun (%)	Mon (%)	Tue (%)	Wed (%)	Thu (%)	Fri (%)	Sat (%)
F	Baoshan	0.55	0.33	0.30	0.23	0.33	0.40	0.39
	Changning	0.63	0.34	0.41	0.64	0.49	0.56	0.57
	Hongkou	0.50	0.41	0.37	0.50	0.49	0.43	0.68
	Huangpu	0.72	0.53	0.62	0.73	0.72	0.62	0.87
	Jingan	0.62	0.54	0.55	0.57	0.46	0.47	0.88
	Minhang	0.37	0.31	0.37	0.28	0.32	0.32	0.48
	Pudong New Area	1.37	1.32	1.03	0.92	1.23	1.69	1.74
	Putuo	0.84	0.50	0.63	0.78	0.57	0.69	0.90
	Xuhui	0.87	0.83	0.60	0.87	0.72	0.81	0.99
	Yangpu	1.08	0.52	0.56	0.62	0.72	0.89	1.22

M	Baoshan	0.37	0.33	0.42	0.51	0.49	0.31	0.18
	Changning	0.46	0.44	0.83	0.52	0.44	0.45	0.84
	Hongkou	0.83	0.52	0.31	0.62	0.51	0.30	0.79
	Huangpu	0.92	0.74	0.44	0.94	0.53	0.82	0.35
	Jingan	1.10	0.80	0.70	0.52	0.60	0.69	1.04
	Minhang	0.58	0.48	0.75	0.43	0.25	0.50	0.35
	Pudong new area	1.54	0.69	1.61	1.62	1.24	0.85	2.03
	Putuo	1.08	0.83	0.60	0.97	0.80	0.65	0.88
	Xuhui	1.39	0.57	1.01	1.04	0.94	1.14	1.10
	Yangpu	0.89	0.73	0.63	1.23	1.13	0.78	1.58

**Table 3:** Gender Differences during week.

Day	Check-in (%)	Female (%)	Male (%)	*d *_*r*_
Sun	16.74	7.56	9.18	0.193
Mon	11.76	5.63	6.13	0.085
Tue	12.74	5.44	7.30	0.291
Wed	14.54	6.15	8.39	0.308
Thu	12.98	6.05	6.92	0.135
Fri	13.37	6.89	6.48	0.062
Sat	17.87	8.73	9.14	0.046

**Table 4:** Gender difference in districts.

Day	Check-in (%)	Female (%)	Male (%)	*d *_*r*_
Baoshan	5.12	2.52	2.60	0.032
Changning	7.64	3.66	3.98	0.085
Hongkou	7.26	3.38	3.88	0.137
Huangpu	9.56	4.81	4.75	0.136
Jingan	9.56	4.11	5.45	0.281
Minhang	5.80	2.45	3.35	0.309
Pudong new area	18.88	9.31	9.58	0.028
Putuo	10.72	4.91	5.81	0.168
Xuhui	12.89	5.70	7.19	0.232
Yangpu	12.57	5.61	6.96	0.216

## Data Availability

The dataset can be acquired from the correspondence upon request.

## References

[1] Alrumayyan N. (2018). Analyzing user behaviors: a study of tips in Foursquare. *5th International Symposium on Data Mining Applications*.

[2] Preston J., Stelter B. (2012). *How Government Officials Are Using Twitter for Hurricane Sandy*.

[3] Hou L., Liu Q., Uddin M., Khattak H., Asshad M. (2021). Spatiotemporal analysis of residents in shanghai by utilizing Chinese microblog Weibo data. *Mobile Information Systems*.

[4] Chatterjee P. (2013). Big data: the greater good or invasion of privacy. *The Guardian*.

[5] Dumbill E. (2012). What is big data: an introduction to the big data landscape (article). *Strata Oreilly*.

[6] Graham M., Shelton T. (2013). Geography and the future of big data, big data and the future of geography. *Dialogues in Human geography*.

[7] Lohr S. (2013). Big data is opening doors, but maybe too many. *New York Times*.

[8] Mayer-Schönberger V., Cukier K. (2013). *Big Data: A Revolution that Will Transform How We Live, Work, and Think*.

[9] Ovadia S. (2013). The role of big data in the social sciences. *Behavioral & Social Sciences Librarian*.

[10] Chai Y. (2012). Review for space-time behavior research: theory frontiers and application in the future. *Progress in Geography*.

[11] Lv Z., Lv S., Feng H., Zhu H., Lv H. (2021). Clinical characteristics and analysis of risk factors for disease progression of COVID-19: a retrospective Cohort Study. *International Journal of Biological Sciences*.

[12] Wang K., Zhang B., Alenezi F., Li S. (2022). Communication-efficient surrogate quantile regression for non-randomly distributed system. *Information Sciences*.

[13] Zhang F., Zhai J., Shen X., Mutlu O., Du X. (2022). POCLib: a high-performance framework for enabling near orthogonal processing on compression. *IEEE Transactions on Parallel and Distributed Systems*.

[14] Gonzalez M. C., Hidalgo C. A., Barabasi A.-L. (2008). Understanding individual human mobility patterns. *Nature*.

[15] Song C., Qu Z., Blumm N., Barabasi A. L. (2010). Limits of predictability in human mobility. *Science*.

[16] Zhu X. (2014). GIS and urban mining. *Resources*.

[17] Cao Z., Wang Y., Zheng W. (2022). The algorithm of stereo vision and shape from shading based on endoscope imaging. *Biomedical Signal Processing and Control*.

[18] Zhang M., Chen Y., Lin J. (2021). A privacy-preserving optimization of neighborhood-based recommendation for medical-aided diagnosis and treatment. *IEEE Internet of Things Journal*.

[19] Kumar A., Singh A. K., Ahmad I. (2022). A novel decentralized blockchain architecture for the preservation of privacy and data security against cyberattacks in healthcare. *Sensors*.

[20] Muhammad Hussain N., Rehman A. U., Othman M. T. B., Zafar J., Zafar H., Hamam H. (2022). Accessing artificial intelligence for fetus health status using hybrid deep learning algorithm (AlexNet-SVM) on cardiotocographic data. *Sensors*.

[21] Kanwal S. (2022). A robust data hiding reversible technique for improving the security in e-health care system. *CMES-COMPUTER MODELING IN ENGINEERING & SCIENCES*.

[22] Ahmad I., Ullah I., Khan W. U. (2021). Efficient algorithms for E-healthcare to solve multiobject fuse detection problem. *Journal of Healthcare Engineering*.

[23] Rehman A. U., Naqvi R. A., Rehman A., Paul A., Sadiq M. T., Hussain D. (2020). A trustworthy siot aware mechanism as an enabler for citizen services in smart cities. *Electronics*.

[24] Khushi M., Shaukat K., Alam T. M. (2021). A comparative performance analysis of data resampling methods on imbalance medical data. *IEEE Access*.

[25] Shaukat K., Luo S., Varadharajan V. (2020). Performance comparison and current challenges of using machine learning techniques in cybersecurity. *Energies*.

[26] Liu X., Zhao J., Li J., Cao B., Lv Z. (2022). Federated neural architecture search for medical data security. *IEEE Transactions on Industrial Informatics*.

[27] Zhang Z., Wang L., Zheng W., Yin L., Hu R., Yang B. (2022). Endoscope image mosaic based on pyramid ORB. *Biomedical Signal Processing and Control*.

[28] Tang Y., Liu S., Deng Y., Zhang Y., Yin L., Zheng W. (2021). An improved method for soft tissue modeling. *Biomedical Signal Processing and Control*.

[29] Liu Y., Tian J., Hu R. (2022). Improved feature point pair purification algorithm based on SIFT during endoscope image stitching. *Frontiers in Neurorobotics*.

[30] Liu S., Yang B., Wang Y., Tian J., Yin L., Zheng W. (2022). 2D/3D multimode medical image registration based on normalized cross-correlation. *Applied Sciences*.

[31] Wixom B., Watson H. (2010). The BI-based organization. *International Journal of Business Intelligence Research*.

[32] Wieder B., Ossimitz M.-L. (2015). The impact of Business Intelligence on the quality of decision making–a mediation model. *Procedia Computer Science*.

[33] Grublješič T., Jaklič J. (2015). Conceptualization of the business intelligence extended use model. *Journal of Computer Information Systems*.

[34] Lv Z., Chen D., Lv H. (2022). Smart city construction and management by digital twins and BIM big data in COVID-19 scenario. *ACM Transactions on Multimedia Computing, Communications, and Applications*.

[35] Ahmad S., Miskon S., Alkanhal T. A., Tlili I. (2020). Modeling of business intelligence systems using the potential determinants and theories with the lens of individual, technological, organizational, and environmental contexts-a systematic literature review. *Applied Sciences*.

[36] Singh Y. S. (2019). Easy designing steps of a local data warehouse for possible analytical data processing. *ADBU Journal of Engineering Technology*.

[37] Santos V., Silva R., Belo O. (2014). Towards a low cost ETL system. *International Journal of Database Management Systems*.

[38] Shaukat K. Student’s performance in the context of data mining.

[39] Latif M. Z. Risk factors identification of malignant mesothelioma: a data mining based approach.

[40] Shaukat K., Zaheer S., Nawaz I. (2015). Association rule mining: an application perspective. *International Journal on Control System and Instrumentation*.

[41] Alam T. M., Shaukat K., Hameed I. A. (2020). An investigation of credit card default prediction in the imbalanced datasets. *IEEE Access*.

[42] Li C., Dong M., Li J. (2022). Efficient medical big data management with keyword-searchable encryption in healthchain. *IEEE Systems Journal*.

[43] Alam T. M., Shaukat K., Hameed I. A. (2021). A novel framework for prognostic factors identification of malignant mesothelioma through association rule mining. *Biomedical Signal Processing and Control*.

[44] Cho E., Myers S. A., Leskovec J. Friendship and mobility: user movement in location-based social networks.

[45] Gao H., Tang J., Liu H. Exploring social-historical ties on location-based social networks.

[46] Lindqvist J. I’m the mayor of my house: examining why people use foursquare-a social-driven location sharing application.

[47] Scellato S. Socio-spatial properties of online location-based social networks.

[48] Zhang J.-D., Chow C.-Y. iGSLR: personalized geo-social location recommendation: a kernel density estimation approach.

[49] Colombo G. B. You are where you eat: foursquare checkins as indicators of human mobility and behaviour.

[50] Li Y. Exploring venue popularity in foursquare.

[51] Lin S. Understanding user activity patterns of the swarm app: a data-driven study.

[52] Shi B., Zhao J., Chen P.-J. (2017). Exploring urban tourism crowding in Shanghai via crowdsourcing geospatial data. *Current Issues in Tourism*.

[53] Gu Z., Zhang Y., Chen Y., Chang X. (2016). Analysis of attraction features of tourism destinations in a mega-city based on check-in data mining—a case study of ShenZhen, China. *ISPRS International Journal of Geo-Information*.

[54] Long Y., Han H., Tu Y., Shu X. (2015). Evaluating the effectiveness of urban growth boundaries using human mobility and activity records. *Cities*.

[55] Wu C., Ye X., Ren F., Du Q. (2018). Check-in behaviour and spatio-temporal vibrancy: an exploratory analysis in Shenzhen, China. *Cities*.

[56] Wu J., Li J., Ma Y. (2019). A comparative study of spatial and temporal preferences for waterfronts in Wuhan based on gender differences in check-in behavior. *ISPRS International Journal of Geo-Information*.

[57] Jia T., Cai C., Li X., Luo X., Zhang Y., Yu X. (2022). Dynamical community detection and spatiotemporal analysis in multilayer spatial interaction networks using trajectory data. *International Journal of Geographical Information Science*.

[58] Pezoa F. Foundations of JSON schema.

[59] Zhou J., Hou Q., Dong W. (2019). Spatial characteristics of population activities in suburban villages based on cellphone signaling analysis. *Sustainability*.

[60] Lichman M., Smyth P. Modeling human location data with mixtures of kernel densities.

[61] Loo B. P., Yao S., Wu J. Spatial point analysis of road crashes in Shanghai: a GIS-based network kernel density method.

[62] Chen X., Li X., Guan J. (2019). Research on the temporal and spatial characteristics of tourist flow in tianchi lake scenic spot based on micro-blog check-in data. *Areal Research and Development*.

[63] Feng Y., Liu Y., Tong X. (2018). Spatiotemporal variation of landscape patterns and their spatial determinants in Shanghai, China. *Ecological Indicators*.

[64] Kotus J., Rzeszewski M., Ewertowski W. (2015). Tourists in the spatial structures of a big Polish city: development of an uncontrolled patchwork or concentric spheres?. *Tourism Management*.

[65] Lei C., Zhang A., Qi Q., Su H., Wang J. (2018). Spatial-temporal analysis of human dynamics on urban land use patterns using social media data by gender. *ISPRS International Journal of Geo-Information*.

[66] Liu Q., Hou L., Shaukat S., Tariq U., Riaz R., Rizvi S. S. (2021). Perceptions of spatial patterns of visitors in urban green spaces for the sustainability of smart city. *International Journal of Distributed Sensor Networks*.

[67] Scellato S. Socio-spatial properties of online location-based social networks.

[68] Wang B., Zhen F., Wei Z., Guo S., Chen T. (2015). A theoretical framework and methodology for urban activity spatial structure in e-society: empirical evidence for Nanjing City, China. *Chinese Geographical Science*.

[69] Ullah H., Wan, Haidery, Khan, Ebrahimpour, Luo (2019). Analyzing the spatiotemporal patterns in green spaces for urban studies using location-based social media data. *ISPRS International Journal of Geo-Information*.

[70] Ullah H., Wan W., Haidery S. A., Khan N. U., Ebrahimpour Z., Muzahid A. A. M. (2020). Spatiotemporal patterns of visitors in urban green parks by mining social media big data based upon WHO reports. *IEEE Access*.

[71] Ebrahimpour Z., Wan, Cervantes, Luo, Ullah (2019). Comparison of main approaches for extracting behavior features from crowd flow analysis. *ISPRS International Journal of Geo-Information*.

[72] Liu Q., Ullah H., Wan W. (2020). Analysis of green spaces by utilizing big data to support smart cities and environment: a case study about the city center of shanghai. *ISPRS International Journal of Geo-Information*.

[73] Ali Haidery S., Ullah H., Khan N. U., Fatima K., Rizvi S. S., Kwon S. J. (2020). Role of big data in the development of smart city by analyzing the density of residents in shanghai. *Electronics*.

[74] Liu Q., Ullah H., Wan W. (2020). Categorization of green spaces for a sustainable environment and smart city architecture by utilizing big data. *Electronics*.

[75] Hou L., Liu Q., Nebhen J., Uddin M., Ullah M., Khan N. U. (2021). Analyzing the check-in behavior of visitors through machine learning model by mining social network’s big data. *Computational and Mathematical Methods in Medicine*.

[76] Heikinheimo V., Tenkanen H., Bergroth C., Jarv O., Hiippala T., Toivonen T. (2020). Understanding the use of urban green spaces from user-generated geographic information. *Landscape and Urban Planning*.

[77] Boeing G. (2021). Spatial information and the legibility of urban form: big data in urban morphology. *International Journal of Information Management*.

[78] Wu W. (1999). City profile: shanghai. *Cities*.

[79] Nam T., Pardo T. A. Conceptualizing smart city with dimensions of technology, people, and institutions.

[80] Zheng W., Xun Y., Wu X., Deng Z., Chen X., Sui Y. (2021). A comparative study of class rebalancing methods for security bug report classification. *IEEE Transactions on Reliability*.

[81] Yang W., Chen X., Xiong Z., Xu Z., Liu G., Zhang X. (2021). A privacy-preserving aggregation scheme based on negative survey for vehicle fuel consumption data. *Information Sciences*.

[82] Khan N. U., Wan W., Yu S., Muzahid A. A. M., Khan S., Hou L. (2020). A study of user activity patterns and the effect of venue types on city dynamics using location-based social network data. *ISPRS International Journal of Geo-Information*.

[83] Khan N. U., Wan W., Yu S. (2020). Spatiotemporal analysis of tourists and residents in Shanghai based on location-based social network’s data from Weibo. *ISPRS International Journal of Geo-Information*.

